# Impact of an Early Childhood Development Intervention on the Mental Health of Female Caregivers: Evidence from a Randomized Controlled Trial

**DOI:** 10.3390/ijerph191811392

**Published:** 2022-09-10

**Authors:** Yu Bai, Reyila Abulitifu, Dan Wang

**Affiliations:** 1 School of Economics, Minzu University of China, Beijing 100081, China; 2 China Institute for Vitalizing Border Areas and Enriching the People, Beijing 100081, China

**Keywords:** female caregivers, mental health, early childhood development intervention

## Abstract

Investing in early childhood development is an effective way to enhance human capital accumulation. Caregivers’ mental health is one of the most important factors influencing children’s development. Previous studies have found that mental health issues in caregivers are widespread all over the world, especially in low- and middle-income countries. In this study, we explored the effects of the “Integrated Program for Early Childhood Development” on the mental health of female caregivers in Southwest China through a randomized intervention trial, with infants aged 5–25 months and their caregivers as the target subjects. The heterogeneity of the effects of different characteristics of the caregivers and the mechanism of the intervention effect were also analyzed. Primary caregivers were provided comprehensive early development interventions for the children in the treatment group via bi-weekly home visiting activities and monthly family group activities. The results showed that the prevalence of depression, anxiety, and stress symptoms among female caregivers in this rural area were 32%, 42%, and 30%, respectively. Whether the child was breastfed, parent’s age, parent’s education level, primary caregiver type, the ratio of the number of months the mother was at home full time to the child’s age, the grandmother’s rearing ability, and the family asset index were the factors influencing the mental health of female caregivers. The intervention significantly increased the proportion of depressive symptoms in 28% of the grandmothers. It significantly reduced the anxiety symptoms of daughters-in-law not from the local town, while the social interactions of both local and non-local daughters-in-law were significantly improved.

## 1. Introduction

Investing in early childhood development is an effective way to enhance human capital accumulation [1,2,3]. The period from 0 to 3 years is sensitive and critical for forming human capital [2,4]. The average return on human capital investment decreases with age, and the highest return occurs between the ages of 0 and 3 years [2,5]. Children’s development during this period has a long-term and significant impact on their future human capital development [6].

Children in rural areas of China aged 0–3 years are seriously lagging in their development [7,8,9,10]. The percentage of children with at least one lag in their development of cognitive, language, motor, and social-emotional skills is 86% [11], and the risk of cognitive, language, motor, and social-emotional delays in children is 53%, 60%, 36%, and 40%, respectively [12].

These high rates of delays are associated with a lack of parental engagement [13,14] and also related to unscientific feeding and parenting practices [15]. The parent–child interaction rate of storytelling, singing children’s songs, and reading books did not exceed 40% in rural China, and only 4% of them had read to their child yesterday [16]. Overall, infant feeding practices are poor in rural China, and grandmothers engage in poorer feeding practices than mothers; for example, grandmothers feed a less diversified diet to children than do mothers [15]. Moreover, fat is still widely associated with health in Chinese culture, particularly among grandparents, and because of this belief, caregivers, who are mostly grandparents, frequently force children to eat a certain amount of food at each meal, regardless of the child’s hunger and satiety feedback [17].

Caregivers’ mental health is also one of the most important factors influencing children’s development, especially when children are young [18,19,20,21]. From childhood to adolescence, children of depressed mothers are more likely to have psychological and psychopathology problems [22,23,24,25,26] and are more likely than children of non-depressed mothers to be underweight, stunted [27], undernourished [20], or have attention deficits [28]. Furthermore, caregivers with poor mental health also have negative impacts on their children in terms of cognitive development, language development, social emotions, adaptability, and behavior [19,24,29,30,31,32,33]. Some 36–46% of children under the age of five are stunted and have slower early cognitive, language, and social-emotional development in low- and middle-income rural areas of West-central China [7]. These delays are positively correlated with negative parenting behavior [16,34]. Caregivers with poor mental health tend to adopt negative parenting behavior [21]. Depressed mothers show more detachment, irritability, hostility, and other negative interactions with their children [35,36]. They are also less likely to engage in stimulating activities such as storytelling, reading, or singing that are beneficial to the child’s development [21]. Moreover, maternal depression has a significant predictive effect on children’s problem behaviors through an increase in parental conflict [37].

Mental health is also essential for female caregivers’ well-being and social development. Mental health problems have a detrimental impact on women’s quality of life [31] and affect progress toward the achievement of several development goals, such as the promotion of gender equality, the empowerment of women, and the reversal of the spread of HIV/AIDS [38]. Mental health is linked to workers’ physical health, household income, and economic output [38,39]. Besides the negative economic impact on individual consumption levels and productivity, poor mental health also brings a societal financial cost that cannot be underestimated, such as the State having to establish a corresponding guarantee fund to improve such a challenging situation [40].

Mental health issues for caregivers are widespread worldwide and are especially prevalent among child caregivers in rural China. The overall prevalence of depression among mothers of young children ranges from 13% to 22% worldwide [41,42], and 23–55% of primary caregivers in developing countries have mental health symptoms [27]. In low- and middle-income countries, the average prevalence of women’s mental disorders is 19.8% postnatally [43,44,45], which means one in five women have post-partum depression [46]. In rural China, 39% of caregivers have at least one type of mental health issue [21], nearly 32% of caregivers suffer depressive symptoms, 42% suffer anxiety symptoms, and 30% suffer stress symptoms [47]. Nearly 40% of the caregivers of children under three years of age in poverty-stricken areas of China have depression symptoms [10,30,48]. Of the depressed sample, the percentage of mild, moderate, severe, and extreme depressive symptoms is 45%, 42%, 9%, and 4%, respectively [31]. Depressed mothers with absent husbands accounted for 33% [48], and grandmother caregivers suffer from higher depressive symptoms than mothers, reaching 30–34% [18,31,49].

There are many factors influencing the mental health of caregivers that have been shown in previous studies. Among them, lower levels of education, low income, and poor housing status associated with poverty are relevant to the mental health of caregivers in developed and developing countries [41]. The factors related to the mental health of caregivers in low- and middle-income countries include lower levels of education, poor household economic status and relationship problems [43,44,50,51], poor health [52], the number of children [53], and the gender of children [44]. The factors affecting caregivers’ mental health in rural China are similar to those found in low- and middle-income countries, including poorer physical health [49], low-income family finances [47], lower educational level [54], and raising more children [10]. A study conducted in rural China discovered six significant causes of caregiver depression: A lack of social support from family and friends, the burden of caregiving, a lack of control and agency within the household, within-family conflict, poverty, and the perception of material wealth as a measure of self-worth [31].

Early childhood development intervention programs have been implemented in many countries [55], with depression, anxiety, and stress being the most critical aspects of caregivers’ mental health [56]. Previous randomized controlled trials of early childhood development intervention programs were designed to increase parental knowledge and engagement as a way to improve child development [7,13], which means improving the attachment between caregivers and children. To achieve this, parenting concepts and principles are conveyed to caregivers, and caregivers are guided to develop positive parenting behavior through home visits and group activities and to provide emotional support via informal interactions [57,58]. Caregivers’ mental health and child development are mediated by parental investment and skills [32].

There is still no consistent conclusion on whether early childhood development intervention programs improve the mental health of caregivers. Some studies have found that early childhood development intervention programs can help caregivers alleviate their mental health issues [20,59,60,61,62,63,64,65]. In these studies, the intervention resulted in a 0.98-point reduction in caregiver depression scores [61] and an 11% reduction in the proportion of caregivers who were depressed [65]. The reduction varied according to program participation, with higher participation resulting in better mental health relief for caregivers [53]. Caregivers who participated in household activities 40–50 times and 25–29 times had 1.84- and 1.06-point reductions in depression scores, respectively, with no significant effect below 25 times [61]. However, other studies have discovered that early childhood development intervention programs only have a short-term effect on caregiver depression [63]. It has even been noted that early childhood development intervention programs have no significant effects on caregivers’ mental health scores or symptoms in the short- or long-term [1,66,67,68,69]. Therefore, more in-depth research evidence is needed on the impact of early childhood development intervention programs on the mental health of caregivers.

The impact of whether a female primary caregiver married locally or elsewhere on mental health is often overlooked, as well as how childhood development intervention programs affect their mental health issues. According to data from the Seventh Population Census of China, inter-provincial migration is carried out by approximately 124 million people, while approximately 250 million people engage in intra-provincial migration (Seventh Population Census, 2021). Marriage migration of females is an important part of migration in China, accounting for between a quarter and a third of all female migration, and the great majority of rural women’s marriage migration takes place from one rural area to another [70]. As a result, there is part of the group of rural migrant daughters-in-law who migrate because of marriage in rural China. As both “immigrants” and “daughters-in-law”, rural migrant daughters-in-law are outsiders to both local social life and the family life of their husband’s families [71] and face difficulties in social and cultural integration, making this group more vulnerable to loneliness [72,73].

Considering the above, we used data from a randomly selected trial of an early childhood development intervention program for children aged 0–3 years in two towns in rural Western China conducted by the National Health Care Commission Cadre Training Center and the Save the Children in 2019, controlling for fixed town effects, child characteristics, and caregiver characteristics, to investigate the current mental health issues of female primary caregivers, influencing factors, and intervention effects.

Our findings first revealed that 32%, 42%, 30%, and 53% of female caregivers of children in the southwest rural area of China had mild or high levels of depression, anxiety, stress, and any one of these symptoms, respectively. Grandmother caregivers have higher proportions of all symptoms than mothers. Second, we discovered the number of children, whether the child was breastfed, the age of the parents, whether the parents had junior high school or higher education, whether the primary caregiver was the mother, whether the grandmother would be able to rear the child, and the family asset index were all significantly related to the mental health of the primary caregivers. Third, the Early Childhood Development Intervention Program had an impact on the proportion of grandmothers’ depressive symptoms, as well as significantly reducing the proportion of anxiety symptoms among daughter-in-law caregivers not from the town. We speculate that this may be related to the intervention improving the increased social interaction of daughters-in-law not from the town. We also found that the intervention program had a greater impact on the social interactions of local daughters-in-law than those not from the town.

The contribution of this research is that we focused on female caregivers, and an early development intervention program was found to increase grandmothers’ depressive symptoms. We also subdivided mother caregivers into local and migrant daughters-in-law and explored the mechanisms of effect by social interaction.

## 2. Methods

### 2.1. Sampling Selection

The data were collected by a cluster-randomized controlled trial conducted in a county of Yunnan Province of Northwestern China. In terms of GDP per capita, Yunnan Province in 2020 had a regional GDP of 2,452,190 billion yuan (Yunnan Provincial Bureau of Statistics, 2021; URL: http://stats.yn.gov.cn/tjsj/tjnj/ (accessed on 23 December 2021), ranking twenty-fifth among the 31 provinces in China (China Statistical Yearbook, 2021; URL: http://www.stats.gov.cn/tjsj./ndsj/ (accessed on 9 October 2021). The county has a total land area of 1484 km^2^ and a population of 500,000, with 14 ethnic groups, including Han, Hui, Yi, Miao, and Buyi, and 21.1% are ethnic minorities (People’s Government of Zhaotong, 2022; URL: http://www.zt.gov.cn/contents/3151/188614.html (accessed on 15 February 2022). The area is typical of poor rural areas of China.

Statistical efficacy was calculated by Optimal Design software. Assuming a statistical power of 80% at the 5% significance level and a correlation coefficient of 0.10 between groups, a minimum of 920 children would need to be sampled. Assuming a 10% sample attrition rate, a minimum of 1012 children needed to be sampled.

The program was implemented in two towns in a north county of Yunnan Province, Southwestern China, consisting of 15 administrative villages and 191 natural villages in total. The program collected samples using a whole-group sampling method, which means that natural villages with one or more eligible children aged 5–25 months were chosen as sample villages. Before the baseline survey, the National Health Commission of the People’s Republic of China collected census data from children aged 5–25 months in the sample towns in March 2019. They identified 1097 eligible children. However, the eligible sample was distributed irregularly across villages.

Finally, the program team conducted a baseline survey of 1024 randomly selected sample households, followed by a follow-up survey of all households that participated in the baseline survey. Our target population was female primary caregivers and their children, with a total sample of 989. This included 494 members of the treatment group from 95 villages and 495 from the control group from 94 villages.

A randomized controlled trial (RCT) whichs an impact evaluation method inwhichch a portion of a qualified target group is randomly assigned to implement an intervention. In contrast, the remaining amount serves as a control group with no intervention. The natural villages in the program are located in remote mountainous areas, far apart from one another, making inter-group information spillover difficult. The experiment was thus randomized at the natural village, and the sample was randomly assigned to either the treatment or control group.

If a household moved out of the sample county, it was no longer followed up for the survey. The selectivity bias was later tested by comparing the differences in the characteristics of the strained and traced samples. Of the 989 caregiver-child dyads enrolled, 94 were lost to follow-up one year after the intervention due to reasons such as relocation, including 51 in the treatment group and 43 in the control group, representing a sample attrition rate of 9.50%. In addition, 74 families were excluded because their caregiver was no longer female at follow-up. Our final sample at follow-up, therefore, comprised 821 caregiver-child dyads, including 410 children and their caregivers in the treatment group and 411 in the control group. The basic individual and family characteristics of the participants are reported in Table 1.

According to the survey data, 78% of the participants in this study were primarily cared for by their mothers, 19% by their grandmothers, and the remaining 3% by their fathers, grandfathers, or other relatives. As a result, most of the children’s primary caregivers in this study were mothers or grandmothers, and the sections that follow are focused on female caregivers.

**Figure 1:**
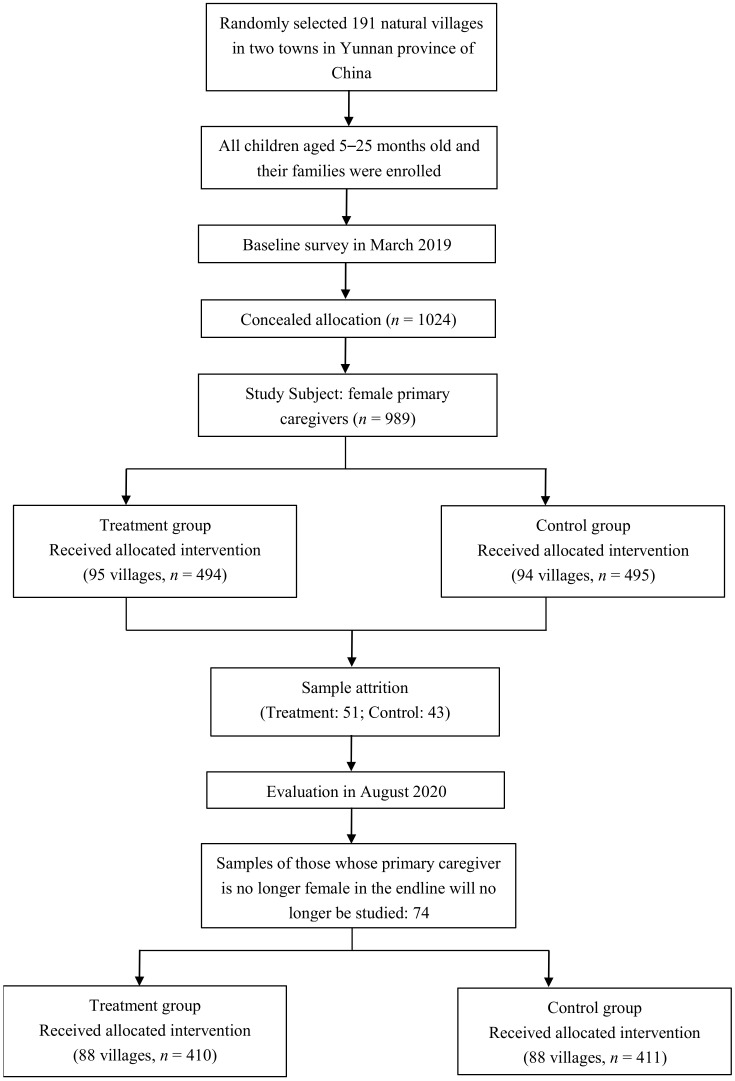
Flowchart of the parenting intervention.

A sample selection flow chart (Figure 1) for the randomized intervention trial in this study is shown below.

### 2.2. Intervention

This program provides monthly group activities and in-home parenting guidance twice a month at intervals of no less than 14 days. Parenting trainers visit the families of the treatment group, providing caregivers and children with parenting training on nutrition and stimulating activities, such as guidance on infant and toddler feeding, child health literacy, children’s picture book reading, and interactive games. In-home tutorials last 45–60 min each time. The family group activities involve three types of activities: interactive book reading, parent–child games, and discussions on parenting issues. The parenting trainers organized monthly group activities for caregivers and children to participate in multiple family activities to help caregivers learn scientific parenting knowledge and to promote the sharing and exchange of experiences in infant and toddler parenting. The activity took place once a month and lasted approximately 60 min.

### 2.3. Data Collection

The data presented in this study were collected in two survey rounds: a baseline survey and a follow-up survey. A total of two phases of large-scale research were conducted to collect sample data. The baseline survey data were collected from 16 March to 2 April 2019, and the follow-up survey was conducted from 1 to 17 August 2020. The intervention should have started in May 2019 and lasted for exactly one year, but due to the coronavirus (COVID-19) pandemic outbreak, Save the Children did not provide intervention from 24 January to 30 April 2020. To meet the goal of providing the intervention for exactly one year, Save the Children resumed the intervention (bi-weekly home visits and monthly group sessions) on 8 May 2020, and ended the intervention on 31 July 2020. By extending the intervention, the children in the treatment group accepted the intervention for 12 months. The follow-up survey started on 1 August 2020 and lasted for approximately two weeks. Ethical approval for this study was granted by Kunming Medical University, Yunnan Province. All subjects provided consent for all data collection activities.

#### 2.3.1. Data Description

Both surveys collected data in two blocks. In the first block, the research team collected detailed information on the child, caregiver, and household characteristics. The second block of the survey collected information on the caregivers’ mental health.

#### 2.3.2. Assessing Caregiver’s Mental Health

This research collected information on the caregivers’ mental health using the Depression Anxiety and Stress Scale-21 (DASS-21). The DASS-21 cannot be interpreted as a tool for direct clinical diagnosis, but it is designed to be a quantitative measure of the severity of depression, anxiety, and stress symptoms. Studies have proved the high construct validity of the DASS-21 and have established the cross-cultural validity of the DASS-21 in China [74,75].

The DASS-21 is a self-report questionnaire consisting of 21 items that measure distress levels over the previous week along three dimensions: depression, anxiety, and stress. It is scored on a 4-point scale from 0 to 3, with 0 representing no compliance at all, 1 representing partial compliance, 2 representing considerable compliance, and 3 representing complete compliance. Scores for each of the three DASS-21 subscales (depression, anxiety, and stress) were derived by totaling the scores for each subscale and multiplying them by 2. The final scores ranged from 0 to 42, with higher scores indicating increasing severity of depression, anxiety, or stress. We used these continuous scores as a measure of the severity of mental health issues among sample caregivers.

In addition, we calculated a binary variable to measure the prevalence of caregivers who were symptomatic of mental health issues (where 1 = symptomatic and 0 = not symptomatic). Scaled scores for the DASS-21 subscales of depression, anxiety, and stress were derived by totaling the scores for each subscale and multiplying them by 2. The severity of the participants’ mental health was divided into the following categories: Normal (0–9 for depression, 0–7 for anxiety, and 0–14 for stress), mild (10–13 for depression, 8–9 for anxiety, and 15–18 for stress), moderate (14–20 for depression, 10–14 for anxiety, and 19–25 for stress), severe (21–27 for depression, 15–19 for anxiety, and 26–33 for stress), and extremely severe (≥28 for depression, ≥20 for anxiety, and ≥34 for stress). A score of DASS ≥10 was used to identify caregivers who have symptoms of depression, a score of DASS ≥8 was used to identify caregivers who have symptoms of anxiety, and a score of DASS ≥15 was used to identify caregivers who have symptoms of stress.

#### 2.3.3. Interactive Parenting Practices

To assess caregiver parenting practices, both positive and negative, we asked the primary caregivers whether or not they had engaged in several interactive practices the previous day: told stories to the baby, read books to the baby, sang songs to the baby, used toys to play with the baby, and the number of times they expressed affection to the baby. In addition, we asked the primary caregivers how often they engaged in the following negative parenting practices: raise their voice or yell at the baby, spank the baby, take away toys from the baby, or do not explain to the baby why his or her behavior is inappropriate.

#### 2.3.4. Child and Household Characteristics

We used a two-dimensional table of family members to collect detailed information on the children, caregivers, and family characteristics. The information for the children included their age, gender (1 = male and 0 = female), whether they were born prematurely (1 = yes and 0 = no), the number of siblings, and whether they were breastfed (1 = yes and 0 = no).

The program also collected information on the characteristics of each household. These include the caregiver’s age and education level (1 = junior high school and above and 0 = below junior high school), whether the mother was the primary caregiver (1 = yes and 0 = no), the ratio of the number of months the mother was at home full time to the child’s age, and whether the parents were away from home (1 = yes and 0 = no). Additionally, regarding whether the grandmother could raise the child (1 = yes and 0 = no), the options of “very healthy”, “healthy”, and “average” mean the grandmother can share the rearing burden, while “unhealthy”, “very unhealthy”, “deceased”, or “other” means the grandmother cannot raise the child. Whether the parents were absent refers to at least one parent being away for three months or more from July 2019 to June 2020. Regarding whether the mother is not from the town (where 1 = yes and 0 = no) (where 1 = yes and 0 = no), we define “daughters-in-law not from the town” as the mother of a caregiver who is not from the town.

To measure the basic situation of the child’s family more precisely, we investigated the family’s income in 2018 and further calculated the household asset value. The family asset value was generated by the internationally used principal component analysis method. We asked the following six questions: Do you have a flush toilet in your home? Do you have a water heater at home? Do you have a computer at home? Do you have Internet access at home? Do you have air conditioning at home? Do you have a car/van at home? The answer options for all questions were 1 = yes and 0 = no.

The primary caregiver’s social interactions consisted of the number of friends seen regularly, the number of times they took the child to see friends in the past month, the number of families the primary caregiver interacted with in the past month, and the number of days the primary caregiver interacted with other families outside of group activities in the past month.

### 2.4. Statistical Analysis

#### 2.4.1. Female Caregivers’ Mental Health Outcomes with Children and Family Characteristics

We used multiple linear regression to analyze the correlations between child characteristics, household characteristics, and the mental health of the child’s primary caregiver with the following econometric model:

(1) $$y_{i}=\beta_{0}+\beta_{1}\times {\mathrm{factor}}_{i}+\varepsilon_{i}\;$$

where $y_{i}\;$indicates the mental health of the child’s primary caregiver with six indicators in three dimensions, including scores of depression, anxiety, and stress, and symptoms of depressive, anxiety, and stress. ${\mathrm{factor}}_{i}$ indicates factors associated with the mental health of the primary caregivers of the children. Among them, the child’s characteristics were the child’s age, gender, whether they were born prematurely, the number of siblings, and whether they were breastfed. The household characteristics include the parent’s age and education level, whether the mother was the primary caregiver, the ratio of the number of months the mother was at home full time to the child’s age, whether the parents were away from home, the grandmother’s rearing ability, whether the mother is not from the town, caregivers’ ethnicity, and the family asset index. We accounted for the clustering at the village level and control for the towns’ fixed effects.

#### 2.4.2. Average Treatment Effect on the Mental Health of Primary Caregivers

To assess the average treatment effect (ATE) of the program on the treatment group, we regressed the intervention variables on the outcome variables, and the model was set up as follows:

(2) $${\;y}_{1\mathrm{is}}=\alpha +\beta \times T_{s}+\theta \times y_{0\mathrm{is}}+\gamma \times X_{\mathrm{is}}+\varepsilon_{\mathrm{is}}$$

where $y_{\mathrm{is}}$ indicates the mental health of the primary caregiver of the child in natural villages at the end-line, and $T_{s}$ is a dummy variable for whether the natural village is in the treatment group, where 1 indicates that the village belongs to the treatment group. The estimates of β are the unbiased estimates of the interventions, as the intervention was randomly assigned. $y_{0\mathrm{is}}$ is the mental health level of the primary caregiver at baseline.

In addition, several control variables were added to the model to increase the validity of the estimates. $X_{\mathrm{is}}$ is a set of control variables for the child, caregiver, and family characteristics, and the scores and symptoms of the caregivers’ mental health at baseline. The child’s characteristics were the child’s age, gender, whether they were born prematurely, the number of siblings, and whether they were breastfed. The household characteristics included the parent’s age and education level, whether the mother was the primary caregiver, the ratio of the number of months the mother was at home full time to the child’s age, whether the parents were away from home, whether the grandmother could raise the child, whether the mother is not from the town, caregivers’ ethnicity, and the family asset index. We accounted for the clustering at the village level, and control for the towns’ fixed effects.

#### 2.4.3. Local Average Treatment Effect on the Mental Health of Caregivers

To assess the local treated average treatment effect (LATE) on the treatment group that substantially participated in the program, we used a quasi-experimental approach with instrumental variables (IVs) and two-stage least squares (2SLS) to estimate the actual impact of the intervention. In this study, the assigned intervention activities were the instrumental variables, and the number of home visiting activities and family group activities received by the children and their primary caregivers were the endogenous variables. The first stage regression equation is as follows:

(3) $$p_{i}=\propto_{1}+\beta_{1}\times T_{i}+\gamma_{1}\times X_{i}+\varepsilon_{i}$$

We used first-stage regression to predict the actual probability of receiving the intervention.

(4) $${\hat{p}}_{i}=\hat{\propto }+\hat{\beta }\times T_{i}+\hat{\gamma }\times X_{i}$$

A second-stage regression was based on Equation (4).

(5) $$y_{1i}=\propto_{2}+\beta_{2}\times {\hat{p}}_{i}+\theta \times y_{0i}+\gamma_{2}\times X_{i}+\varepsilon_{i}$$

Among them, $p_{i}$ is the number of times that the children and caregivers in the treatment group received home visiting activities or participated in family group activities; ${\hat{p}}_{i}$ is the predicted value of the explanatory variable${\;p}_{i}$; $T_{i}$ indicates whether it is an experimental group, which is an instrumental variable; $y_{1i}$ refers to the indicators of mental health for caregivers at the end-line; $y_{0i}$ refers to indicators of mental health for caregivers at baseline$;{\;X}_{\mathrm{is}}$ is a set of control variables for the child, household characteristics, and the scores and symptoms of caregivers’ mental health at baseline$;{\;\beta }_{2}$ is the unbiased estimator of the actual intervention effect. We accounted for clustering at the village level and controlled for the towns’ fixed effects.

#### 2.4.4. Heterogeneous Effects of the Intervention on the Mental Health of Female Caregivers

To investigate the heterogeneous effects of the intervention on the child and household with different characteristics, we added a cross-section of variables indicating the child and household characteristics versus intervention dummy variables to the model. The estimated model is as follows:

(6) $$y_{1\mathrm{is}}=\alpha +\beta \times T_{s}+\delta \times H+\eta \times T_{s}\times H+\theta \times y_{0\mathrm{is}}+\gamma \times X_{\mathrm{is}}+\varepsilon_{\mathrm{is}}$$

where the coefficient β of the cross-term represents the effect of heterogeneity, and H represents the variables of different characteristic groups. We accounted for clustering at the village level and controlled for the towns’ fixed effects.

## 3. Results

### 3.1. Child and Household Characteristics

Table 1 shows the basic socioeconomic and demographic characteristics of these two blocks. In the all-female samples at baseline, there were 989 observations. When looking at the child’s characteristics, the data show that the average age of the children was 15.23 months, and over half (52%) were male. Around 5% of the sample children were born prematurely, each child in the sample had an average of one sibling, and 95% of the children were breastfed.

In terms of caregiver characteristics, the average ages of the children’s mothers and fathers were 26 and 29 years old, respectively. More than 51% of the mothers had junior high school education or higher, and 46% of the fathers had junior high school education or higher. Moreover, 81% of the primary caregivers at baseline were mothers, 37% of whom were daughters-in-law not from the town. The ratio of the number of months the mother was at home full time to the child’s age was 91%. A total of 57% of the children had either parent absent for three months or more, 65% of the grandmothers were capable of raising the child, and 92% of the primary caregivers were Han. There were no significant differences in sample characteristics between the treatment and control groups, except for the gender of the children. The data show that the prevalence of depression, anxiety, and stress symptoms in the primary caregivers was 33%, 43%, and 31%, respectively.

### 3.2. Balance

Table 2 shows that the intervention had no significant effect on sample attrition. We conducted a balance test of the remaining sample after both attrition and exclusion (Appendix A, Table A1). The results show no significant differences between the two groups in any of the 18 variables, except the ethnicity and depression symptoms of the primary caregivers. The raw scores and symptoms for depression, anxiety, and stress at baseline for primary caregivers were added as control variables in the regression model.

### 3.3. Prevalence of Caregiver Mental Health Issues

**Figure 2:**
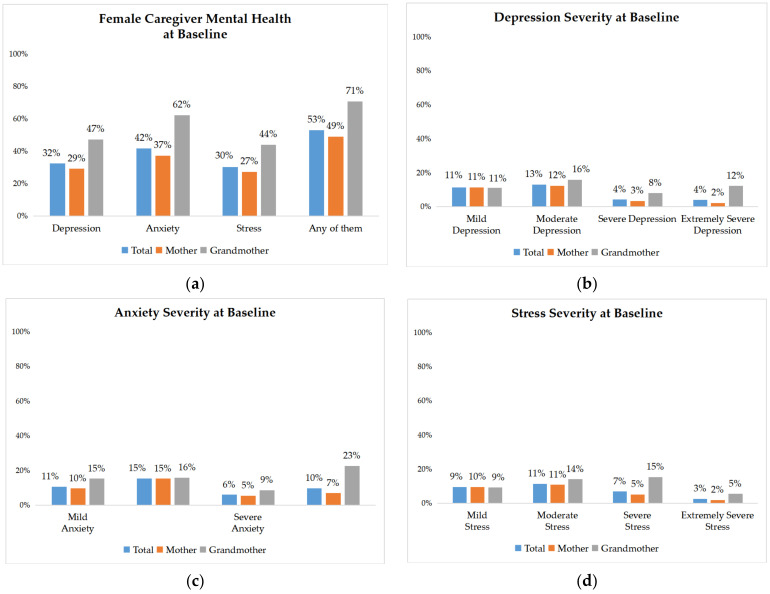
Female caregivers’ mental health characteristics at baseline: (**a**) Female caregivers’ overall mental health at baseline; (**b**) depression severity at baseline; (**c**) anxiety severity at baseline; (**d**) stress severity at baseline.

Overall, a large share of the female primary caregivers in our sample had symptoms of depression, anxiety, or stress (Figure 2). Specifically, 32% of the primary caregivers scored above the threshold for depressive symptoms, 42% scored above the threshold for anxiety symptoms, 30% scored above the threshold for stress symptoms, and 53% suffered from any of the above. A study conducted in a rural area of Sichuan found that the prevalence of depression symptoms among rural Chinese women was 12% [76], and it was 20% lower compared to rural women in Yunnan.

When mental health severity levels were classified as mild, moderate, severe, and extremely severe, it was found that most mental health symptoms in the sample area were mainly concentrated in the mild and moderate ranges, with grandmothers having a largely higher proportion of mental health symptoms than mothers (Figure 2). This is consistent with previous research findings that grandmother caregivers are more likely than mothers to have depressive symptoms as primary caregivers [31,49]. The percentage of extreme anxiety symptoms among grandmothers was 23%.

### 3.4. Trends in Mental Health over Time for Female Caregivers in the Control Group

**Figure 3:**
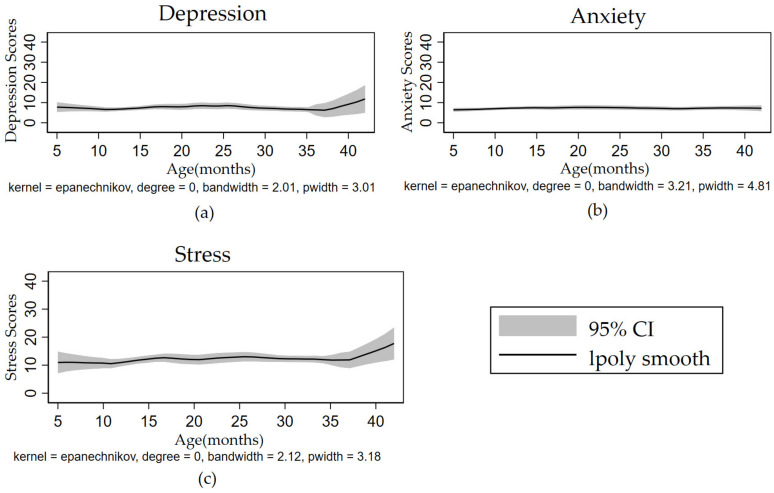
Trends in mental health over time for female caregivers in the control group: (**a**) Depression; (**b**) Anxiety; (**c**) Stress.

The control group did not receive the intervention. Figure 3 shows the trend of the mental health index scores of the infant’s primary caregiver with the infant’s age. There was no significant trend in primary caregiver anxiety scores with increasing child age. However, the depression and stress scores tended to increase with increasing child age, especially in children of approximately 38 months.

### 3.5. Female Caregivers’ Mental Health Outcomes with the Children and Family Characteristics of the Study Sample

Childhood factors related to mental health affecting primary caregivers were analyzed (Table 3). The analysis showed that whether or not the child was breastfed were significantly and negatively associated with the caregiver’s anxiety. In contrast, other studies have shown that parenting multiple children is positively associated with caregivers’ mental health problems [10,20].

In terms of the caregivers and family characteristics, maternal age, the mother being the primary caregiver, and the grandmother’s ability to share the parenting burden are significantly and negatively associated with caregivers’ mental health problems (e.g., depression, anxiety, and stress) (Table 3). It is possible that the older the mother is, the more patience and experience she has in caring for children, which in turn is beneficial to mental health development. When the mother, as the primary caregiver, does not have to be separated from the baby and shares the parenting burden with the grandmother, this contributes to the caregiver’s mental health. The father’s education and family asset index were negatively associated with depression and anxiety. Caregivers in a better family situation face fewer resource constraints, which may be associated with caregivers’ psychological well-being. In previous studies, poorer physical health [49], poor household finances [47], and social support [31,51,76] were factors associated with the level of mental health of the child’s primary caregivers.

### 3.6. Intervention Effect on Female Caregivers’ Mental Health

#### 3.6.1. Female Caregivers’ Mental Health before and after Intervention

**Figure 4:**
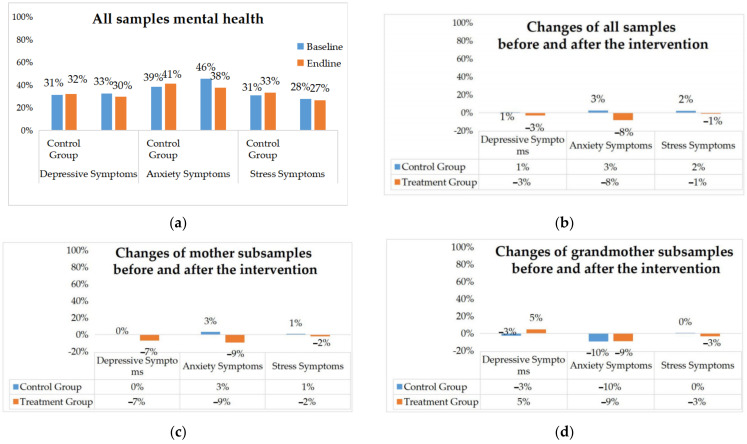
Female caregivers’ mental health before and after the intervention: (**a**) All samples’ mental health; (**b**) changes in all samples before and after the intervention; (**c**) changes in mother subsamples before and after the intervention; (**d**) changes in grandmother subsamples before and after the intervention.

Figure 4 shows that after the one-year intervention period, the primary caregivers in the treatment group experienced 3%, 8%, and 1% reductions in depressive, anxiety, and stress symptoms, respectively, while none of the three indicators decreased in the control group. Specifically, by caregiver type, depression, anxiety, and stress decreased for the mothers in the follow-up compared to the baseline period, and for the grandmothers, depression increased by 5% and anxiety and stress decreased.

#### 3.6.2. Program Average Treated Effect on Caregivers’ Mental Health (ITT)

The multiple regression results indicate that the intervention significantly increased the proportion of depressive symptoms of the grandmothers by 28%, which was significant at the 5% level of statistical significance. There was no significant effect of the intervention on other indicators (Table 4).

#### 3.6.3. Local Average Treatment Effect on the Mental Health of the Caregivers

**Figure 5:**
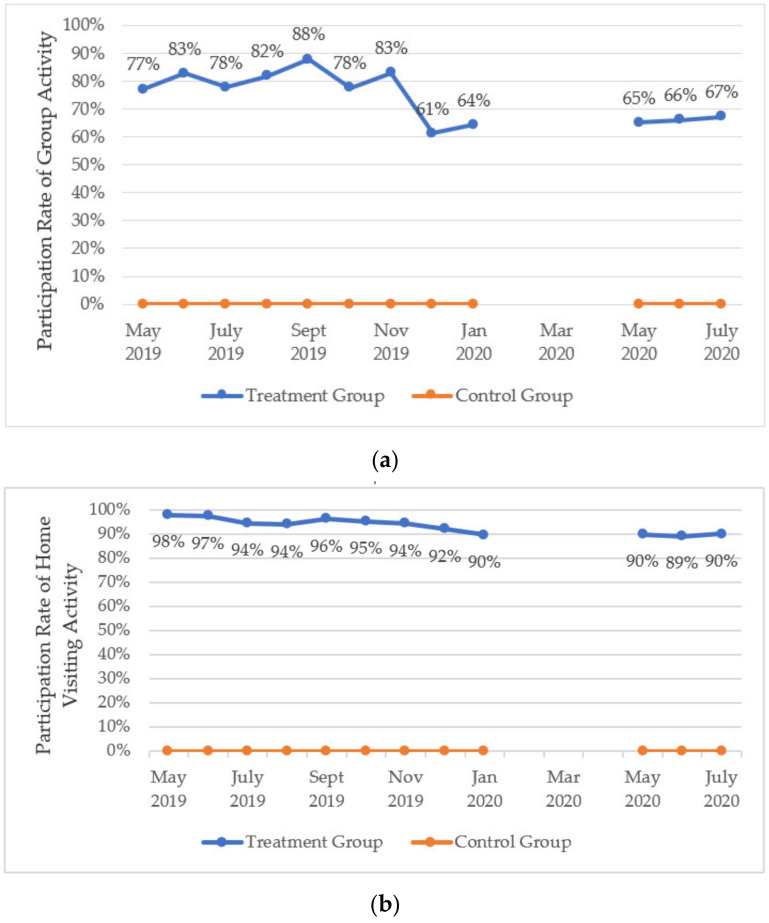
Participation rate of intervention activity: (**a**) Participation rate of group activity; (**b**) Participation rate of home visiting activity.

In the randomized intervention trial, the samples assigned to the treatment group may not all have received the intervention, or may not all have received the intervention at the same frequency, which may have affected the intervention effect, so the effect at different intervention frequencies needs to be analyzed. As shown in Figure 5, none of the samples in the control group in this study received the intervention, and there was no sample contamination.

The compliance with the randomized intervention trial in this study performed well, with an average monthly participation rate of 77.5% in the treatment group for group activities and an average monthly participation rate of 93.3% in the home visiting activities. During the 12 months of the program, the intervention frequency of home visiting and family group activities was once every two weeks and once every month, respectively, so the children in the treatment group should have participated in 24 home visiting service activities and 12 group activities. The mean number of times the treatment group participated in the home visiting and group activities in this study was approximately 22 and 9, respectively. It can be seen that the children in the intervention group did not always participate in the intervention activities. Specifically, 272 primary caregivers participated in group activities nine times or more, accounting for 66.34% of the total sample for the intervention group. As for the participation rate of home visiting activities, 227 primary caregivers, who accounted for 55.37% of the total sample in the treatment group, received home visiting 22 times or more. According to research in China’s Yunnan and Hebei provinces, on average, 15.3 visits out of the 24 planned visits took place [13]. The household and group activity participation rates in an early childhood intervention study conducted in an Indian region were 75% and 51%, respectively [77].

The local average treatment effect analysis results showed that higher participation in group and home visiting activities had a greater negative impact on the grandmothers’ mental health (Appendix A, Table A2, Table A3 and Table 5). Participation in group activities and simultaneous participation in group activities and home visiting had the same impact on the grandmothers’ mental health, significantly increasing grandmother’s depressive symptoms by 44% (5% significance level).

#### 3.6.4. Heterogeneous Effects of the Intervention on the Mental Health of the Female Caregivers

Table 6 shows that there was heterogeneity in the effects of the intervention on the children and caregivers with different characteristics. In terms of the child characteristics, the primary caregivers were more likely to be stressed the younger the child was. In terms of the primary caregiver’s type, the prevalence of depressive symptoms was significantly lower when the caregiver was the mother and higher when the mother was more educated (both at the 5% significance level).

#### 3.6.5. Program Effect on Mental Health of Daughters-in-Law Not from the Town

Based on the heterogeneity analysis in Table 6, we further divided the mothers into town and non-town subsamples based on the median response to the question, “Where is the mother from?”. A total of 797 daughters-in-law were primary caregivers in the base period, including 257 who were not from the town (32.25%) and 465 who were from the town. There were no significant differences between the non-local daughter-in-law caregivers (with 30%, 38%, and 26% showing symptoms of depression, anxiety, and stress, respectively) and the local daughter-in-law caregivers (with 29%, 38%, and 28% showing symptoms of depression, anxiety, and stress, respectively).

Table 7 shows that the intervention significantly improved the depressive symptoms in the daughters-in-law not from the town. Specifically, there was a 15% decrease (5% significance level) in anxiety symptoms for those daughters-in-law not from the town, with no significant effect on the other symptoms.

As shown in Table 8, an analysis of the social interactions between the local and non-local daughters-in-law revealed that the mothers, whether local or not, regularly met with approximately three friends during the baseline and interacted with an average of four families in the past month at the follow-up. The non-local daughters-in-law took their children to see their friends around five times in the past month at baseline, which was significantly higher than that of the local daughters-in-law by 0.24 times (1% significance level). The local daughters-in-law interacted with other families for approximately 11 days in the past month outside of group activities, which was significantly higher than that of those daughters-in-law not from the town by around 3 days (10% significance level).

In terms of the impact of the intervention on the social interaction between the non-local and local daughters-in-law, the intervention significantly increased the number of days that the non-local and local daughters-in-law interacted with other households outside of group activities in the past month, by approximately 13 and 18 days, respectively (Table 8). The intervention had a greater impact on the social interactions of the local daughters-in-law, probably because they have a wider social network and more friends in the area than their non-local counterparts. The intervention was not found to have a significant effect on the “number of friends seen regularly”, “number of times you took your child to see friends in the past month”, or “number of families communicated with in the past month.”

### 3.7. Mechanistic Analysis of the Intervention Effect on the Mental Health Issues of the Primary Caregiver

Although the intervention did not have an overall mitigating effect on the mental health of the female caregivers, the project had a significant impact on the three dimensions of family support, parenting behavior, and social interaction of the female primary caregivers (Table 9). Specifically, there was a significant increase of 0.06 in mutual support between family members, indicating that the intervention had a positive impact on improving the family atmosphere. The negative parenting behavior of the caregiver, “hitting the baby yesterday”, decreased significantly by 13%, while the positive parenting behavior of the caregiver, “explaining to the baby why his behavior was inappropriate”, decreased significantly by 6% (5% significance level). This may be due to a reduction in punitive behaviors such as hitting the baby as the primary caregiver learns scientific feeding concepts during the intervention. However, the lack of proper parenting knowledge, which resulted in the inability to carefully explain to the baby correctly and reasonably during discipline time, may also have affected the caregivers’ mental health.

## 4. Conclusions and Discussion

By analyzing the effect of the intervention on the mental health concerns of primary caregivers, 32%, 42%, and 30% of the female caregivers of children in the southwest rural area of China reported symptoms of depression, anxiety, and stress, respectively, and 53% reported symptoms of any of these conditions. Such symptoms were worse when the primary caregiver was the grandmother compared to when the mother was the primary caregiver. Many studies have found no significant effect of early childhood interventions on the mental health of caregivers [1,66,69], and our study found a similar finding for the mental health of maternal primary caregivers. However, a study of the rural Chinese caregivers’ mental health found no significant effect of a center-based early childhood development intervention on grandmothers’ mental health, and our study provided intervention via home visiting activities and family group activities significantly increased the depressive symptoms of grandmother caregivers by 28%, possibly due to the need for grandmothers to learn new knowledge and to learn about previous unscientific parenting behaviors. The early childhood development intervention also significantly reduced the prevalence of anxiety symptoms of daughter-in-law caregivers who are not from the town.

This study has two possible contributions. Firstly, few studies have focused on the mental health of the out-of-town mother caregiver. We thoroughly divided female caregivers into different subsamples, namely mother caregivers and grandmothers. Secondly, this study analyzed the causal relationship between female caregivers’ mental health and early childhood development intervention in an ethnic area of rural southwest China and attempted to explore the mechanisms of the intervention program’s impact on female caregivers’ mental health in terms of family support, parenting behaviors, parenting burden, and social interactions.

Based on the study results, we present the following suggestions to alleviate the mental health problems of female caregivers in rural areas of China. First, family members should offer more care and support to child caregivers in their lives to minimize the emergence of mental health concerns such as depression, anxiety, and stress and prevent their negative effects on children. Second, government and women federations at all levels should play a more important role in protecting women’s mental health rights and interests and in improving women’s mental health, and at the same time, policies should be formulated with full consideration of the characteristics of caregivers with mental health risks, providing more social support and input to sensitive groups (such as grandmother caregivers) and providing them with scientific parenting advice and methods so as to improve women’s mental health and provide a good environment for children to grow up in. Consideration should also be given to integrating experts and practitioners with relevant knowledge and experience to form a female psychological crisis intervention center to provide timely and necessary assistance to female caregivers with symptoms of mental health problems so that they can alleviate various emotional stresses, learn to face social reality positively, resolve distress, and reduce the occurrence of crisis events and their negative effects on children.

## Figures and Tables

**Table 1:** Descriptive statistics.

	Full Sample	Treatment Group	Control Group	Difference: (2)−(3)
	Mean	Mean	Mean	*p*-Value
	(SD)	(SD)	(SD)	
	(1)	(2)	(3)	(4)
Child Characteristics				
Age (in months) (*n* = 989)	15.23	15.22	15.24	0.94
	(5.59)	(5.74)	(5.44)	
Male (1 = yes) (*n* = 989)	0.52	0.55	0.48	0.04
	(0.50)	(0.50)	(0.50)	
Premature (1 = yes) (*n* = 989)	0.05	0.04	0.06	0.24
	(0.22)	(0.20)	(0.23)	
Number of siblings (*n* = 989)	1.01	0.99	1.03	0.48
	(0.94)	(0.91)	(0.97)	
Breastfed (1 = yes) (*n* = 989)	0.95	0.95	0.96	0.76
	(0.21)	(0.22)	(0.21)	
Household Characteristics				
Mother’s age (*n* = 989)	26.55	26.79	26.31	0.17
	(5.61)	(5.77)	(5.44)	
Father’s age (*n* = 982)	29.64	29.56	29.71	0.65
	(5.37)	(5.33)	(5.41)	
Mother’s education level (1 = 9 years or higher) (*n* = 981)	0.51	0.51	0.51	0.97
	(0.50)	(0.50)	(0.50)	
Father’s education level (1 = 9 years or higher) (*n* = 980)	0.46	0.48	0.44	0.18
	(0.50)	(0.50)	(0.50)	
Primary caregiver (1 = mother) (*n* = 989)	0.81	0.81	0.80	0.64
	(0.40)	(0.39)	(0.40)	
The ratio of the number of months the mother was at home full time to the child’s age (*n* = 989)	0.91	0.92	0.91	0.54
	(0.21)	(0.21)	(0.22)	
Daughter-in-law not from the town (1 = yes) (*n* = 894)	0.37	0.37	0.37	0.93
	(0.48)	(0.48)	(0.48)	
Parents away from home (1 = yes) (*n* = 883)	0.57	0.56	0.57	0.76
	(0.50)	(0.50)	(0.50)	
Grandmother’s rearing ability (1 = yes) (*n* = 989)	0.65	0.64	0.65	0.82
	(0.48)	(0.48)	(0.48)	
Caregiver’s ethnicity (1 = Han) (*n* = 989)	0.92	0.94	0.91	0.10
	(0.27)	(0.24)	(0.29)	
Family asset index (*n* = 989)	–0.00	0.02	–0.02	0.51
	(1.13)	(1.16)	(1.09)	
Caregiver’s Mental Health				
Depression symptoms (1 = yes) (*n* = 988)	0.33	0.33	0.33	0.89
	(0.47)	(0.47)	(0.47)	
Anxiety symptoms (1 = yes) (*n* = 989)	0.43	0.44	0.41	0.26
	(0.49)	(0.50)	(0.49)	
Stress symptoms (1 = yes) (*n* = 989)	0.31	0.29	0.33	0.18
	(0.46)	(0.45)	(0.47)	

Data source: Authors’ survey. Note: The family asset index was generated by the internationally used principal component analysis method based on the following six questions: Do you have a flush toilet in your home? Do you have a water heater at home? Do you have a computer at home? Do you have Internet access at home? Do you have air conditioning at home? Do you have a car/van at home? (The answer options for all questions were 1 = yes, 0 = no).

**Table 2:** Balanced test of the intervention on whether the sample was attrited.

Variables	Whether the Sample Was Attrited (1 = Yes)
	(1)
Treatment (1 = yes)	0.02
	(0.02)
Contents	Yes
Adj. *R*^2^	−0.00
*n*	989

Data source: Authors’ survey. Note: The numbers in parenthesis are standard errors.

**Table 3:** Female caregivers’ mental health outcomes with the children and family characteristics.

	Depression Scores (0–42)	Depressive Symptoms (1 = Yes)	Anxiety Scores (0–42)	Anxiety Symptoms (1 = Yes)	Stress Scores (0–42)	Stress Symptoms (1 = Yes)
	(1)	(2)	(3)	(4)	(5)	(6)
Age (months)	0.04	−0.00	0.05	0.00	0.03	−0.00
	(0.05)	(0.00)	(0.05)	(0.00)	(0.05)	(0.00)
Male (1 = yes)	−0.35	0.02	−0.19	0.02	−0.43	−0.00
	(0.47)	(0.03)	(0.44)	(0.03)	(0.55)	(0.03)
Premature (1 = yes)	1.33	0.04	1.74	0.01	2.30	0.04
	(1.41)	(0.07)	(1.35)	(0.08)	(1.76)	(0.08)
Number of siblings	−0.30	−0.01	−0.50	0.00	−0.33	−0.00
	(0.27)	(0.02)	(0.26)	(0.02)	(0.31)	(0.02)
Breastfed (1 = yes)	−1.25	−0.08	−1.49	−0.20 **	−0.61	−0.03
	(1.53)	(0.09)	(1.30)	(0.08)	(1.82)	(0.10)
Mother’s age	−0.15 **	−0.01	−0.18 **	−0.01 **	−0.16	−0.01 **
	(0.07)	(0.00)	(0.08)	(0.00)	(0.08)	(0.00)
Father’s age	0.10	0.00	0.12	0.01 **	0.06	0.01
	(0.08)	(0.01)	(0.08)	(0.00)	(0.08)	(0.00)
Father’s education level (1 = junior high school and above)	−2.09 **	−0.10 **	−1.57 **	−0.11 **	−0.95	−0.07 **
	(0.59)	(0.04)	(0.58)	(0.04)	(0.71)	(0.03)
Mother’s education level (1 = junior high school and above)	0.12	−0.02	0.07	0.07	1.11	0.07
	(0.75)	(0.04)	(0.73)	(0.04)	(0.82)	(0.04)
Primary caregiver (1 = mother)	−3.31 **	−0.16 **	−4.55 **	−0.22 **	−4.58 **	−0.19 **
	(0.93)	(0.05)	(0.88)	(0.05)	(0.87)	(0.05)
The ratio of the number of months the mother was at home full time to the child’s age	1.62	0.04	1.44	−0.00	2.99	0.12
	(1.26)	(0.08)	(1.03)	(0.09)	(1.58)	(0.10)
Daughter-in-law not from the town (1 = yes)	0.47	0.01	−0.08	−0.00	−0.06	−0.00
	(0.56)	(0.03)	(0.53)	(0.03)	(0.65)	(0.03)
Parents away from home (1 = yes)	0.10	0.01	−0.53	−0.03	−0.38	−0.04
	(0.58)	(0.04)	(0.58)	(0.04)	(0.64)	(0.03)
Grandmother’s rearing ability (1 = yes)	−1.10	−0.08 **	−2.17 **	−0.13 **	−2.37 **	−0.14 **
	(0.56)	(0.03)	(0.63)	(0.04)	(0.71)	(0.04)
Caregivers’ ethnicity (1 = Han)	−1.81	−0.08	−1.09	−0.01	−0.99	−0.00
	(1.14)	(0.05)	(1.21)	(0.07)	(1.31)	(0.05)
Family asset index	−0.80 **	−0.02	−0.44	−0.01	−0.09	0.01
	(0.34)	(0.02)	(0.24)	(0.01)	(0.27)	(0.02)
Adj. *R*^2^	0.08	0.04	0.09	0.07	0.05	0.04
*n*	872	872	873	873	873	873

Data source: Authors’ survey. Note: We controlled for the towns’ fixed effects. All standard errors account for clustering at the village level. ** *p* < 0.05.

**Table 4:** Program average treated effect on the caregivers’ mental health.

	Depression Scores (0–42)	Depressive Symptoms (1 = Yes)	Anxiety Scores (0–42)	Anxiety Symptoms (1 = Yes)	Stress Scores (0–42)	Stress Symptoms (1 = Yes)
	(1)	(2)	(3)	(4)	(5)	(6)
	Panel A: All samples					
Treatment (1 = yes)	0.06	−0.03	0.04	−0.04	−0.05	−0.05
	(0.46)	(0.03)	(0.56)	(0.04)	(0.57)	(0.03)
Adj. *R*^2^	0.09	0.04	0.10	0.07	0.09	0.05
*n*	801	801	802	802	802	802
	Panel B: Mother subsamples					
Treatment (1 = yes)	−0.59	−0.07	−0.45	−0.06	−0.09	−0.06
	(0.47)	(0.04)	(0.50)	(0.04)	(0.55)	(0.04)
Adj. *R*^2^	0.12	0.05	0.10	0.07	0.08	0.04
*n*	562	562	562	562	562	562
	Panel C: Grandmother subsamples					
Treatment (1 = yes)	2.93	0.28 **	3.80	0.09	3.40	0.09
	(1.96)	(0.11)	(2.84)	(0.13)	(2.08)	(0.11)
Adj. *R*^2^	−0.01	0.10	0.03	0.08	0.22	0.12
*n*	88	88	89	89	89	89
Controls	Yes	Yes	Yes	Yes	Yes	Yes
Towns’ Fixed Effects	Yes	Yes	Yes	Yes	Yes	Yes

Data source: Authors’ survey. Note: The controls included the child’s age, gender, premature birth, number of siblings, breastfeeding, age of parents, parent’s education level, whether the mother was the primary caregiver, the ratio of the number of months the mother was at home full time to the child’s age, whether the daughter-in-law was not from the town, whether the parents were away from home, the grandmother’s rearing ability, caregivers’ ethnicity, and the family asset index. We also controlled separately for the level of mental health of the caregivers at baseline and the towns’ fixed effects. All standard errors account for clustering at the village level. ** *p* < 0.05. When regressions were performed on subsamples, whether the mother was the primary caregiver was omitted.

**Table 5:** Local average treatment effect on the mental health of the caregivers who participated in group activities nine times or more and also participated in home visiting 22 times or more.

	Depression Scores (0–42)	Depressive Symptoms (1 = Yes)	Anxiety Scores (0–42)	Anxiety Symptoms (1 = Yes)	Stress Scores (0–42)	Stress Symptoms (1 = Yes)
	(1)	(2)	(3)	(4)	(5)	(6)
	Panel A: All samples					
Treatment (1 = yes)	0.14	−0.06	0.09	−0.09	−0.12	−0.11
	(1.00)	(0.07)	(1.22)	(0.08)	(1.23)	(0.07)
Adj. *R*^2^	0.09	0.05	0.10	0.06	0.09	0.06
*n*	801	801	802	802	802	802
	Panel B: Mother subsamples					
Treatment (1 = yes)	−1.30	−0.15	−0.99	−0.12	−0.20	−0.12
	(1.04)	(0.09)	(1.09)	(0.09)	(1.20)	(0.08)
Adj. *R*^2^	0.12	0.05	0.10	0.06	0.08	0.05
*n*	562	562	562	562	562	562
	Panel C: Grandmother subsamples					
Treatment (1 = yes)	4.70	0.44 **	6.04	0.15	5.46	0.14
	(2.84)	(0.16)	(3.87)	(0.17)	(2.99)	(0.16)
Adj. *R*^2^	−0.06	−0.03	0.02	0.09	0.15	0.09
*n*	88	88	89	89	89	89
Controls	Yes	Yes	Yes	Yes	Yes	Yes
Towns’ Fixed Effects	Yes	Yes	Yes	Yes	Yes	Yes

Data source: Authors’ survey. Note: The controls included the child’s age, gender, premature birth, number of siblings, breastfeeding, age of parents, parent’s education level, whether the mother was the primary caregiver, the ratio of the number of months the mother was at home full time to the child’s age, whether the daughter-in-law was not from the town, whether the parents were away from home, the grandmother’s rearing ability, caregivers’ ethnicity, and the family asset index. We also controlled separately for the level of mental health of the caregivers at baseline and the towns’ fixed effects. All standard errors account for clustering at the village level. ** *p* < 0.05. When regressions were performed on the subsamples, whether the mother was the primary caregiver was omitted.

**Table 6:** Heterogeneous effects of the intervention on the mental health of the female caregivers.

	Depression Scores (0–42)	Depressive Symptoms (1 = Yes)	Anxiety Scores (0–42)	Anxiety Symptoms (1 = Yes)	Stress Scores (0–42)	Stress Symptoms (1 = Yes)
	(1)	(2)	(3)	(4)	(5)	(6)
Panel A: Child age (in months)						
Treatment (1 = yes)	−1.88	−0.18 **	−0.99	−0.16	−2.49	−0.24 **
	(1.19)	(0.08)	(1.28)	(0.10)	(1.50)	(0.09)
Child (in months)	−0.06	−0.00	−0.02	−0.00	−0.05	−0.00
	(0.05)	(0.00)	(0.06)	(0.00)	(0.07)	(0.00)
Treat × Child age	0.12	0.01	0.07	0.01	0.16	0.01 **
	(0.07)	(0.01)	(0.08)	(0.01)	(0.09)	(0.01)
Adj. *R*^2^	0.09	0.05	0.10	0.07	0.09	0.06
*n*	801	801	802	802	802	802
Panel B: Number of child’s siblings						
Treatment (1 = yes)	0.47	0.04	−0.22	−0.05	0.15	−0.03
	(0.73)	(0.05)	(0.80)	(0.06)	(0.84)	(0.05)
Number of child’s siblings	0.59	0.04	0.08	0.00	0.03	0.04
	(0.39)	(0.02)	(0.29)	(0.02)	(0.42)	(0.03)
Treat × Number of child’s siblings	−0.43	−0.06	0.20	0.01	−0.23	−0.02
	(0.54)	(0.03)	(0.47)	(0.04)	(0.47)	(0.03)
Adj. *R*^2^	0.08	0.05	0.10	0.07	0.08	0.05
*n*	801	801	802	802	802	802
Panel C: Whether the child was breastfed (1 = yes)						
Treatment (1 = yes)	2.07	0.01	4.75	0.18	1.56	0.01
	(3.99)	(0.19)	(2.69)	(0.16)	(3.43)	(0.15)
Whether the child was breastfed (1 = yes)	−1.91	0.03	−0.31	−0.00	−1.47	−0.01
	(3.20)	(0.15)	(1.42)	(0.11)	(2.66)	(0.11)
Treat × Whether the child was breastfed	−2.13	−0.04	−4.92	−0.23	−1.72	−0.07
	(4.00)	(0.19)	(2.69)	(0.16)	(3.44)	(0.15)
Adj. *R*^2^	0.08	0.04	0.10	0.07	0.08	0.05
*n*	801	801	802	802	802	802
Panel D: Primary caregiver (1 = mother)						
Treatment (1 = yes)	0.89	0.15	1.10	−0.03	0.49	−0.02
	(1.43)	(0.08)	(1.85)	(0.09)	(1.45)	(0.08)
Primary caregiver (1 = mother)	−0.70	0.08	−0.55	−0.04	−0.05	0.01
	(1.04)	(0.06)	(1.20)	(0.09)	(1.09)	(0.07)
Treat × Primary caregiver	−1.05	−0.22 **	−1.32	−0.02	−0.71	−0.04
	(1.50)	(0.09)	(1.84)	(0.10)	(1.52)	(0.09)
Adj. *R*^2^	0.08	0.05	0.10	0.07	0.08	0.05
*n*	801	801	802	802	802	802
Panel E: Mother’s education level (1 = junior high school and above)						
Treatment (1 = yes)	−0.10	−0.09 **	0.46	−0.04	−0.66	−0.09
	(0.73)	(0.05)	(0.84)	(0.05)	(0.75)	(0.04)
Mother’s education level (1 = junior high school and above)	−0.86	−0.07	0.00	−0.05	−0.83	−0.03
	(0.77)	(0.05)	(0.83)	(0.05)	(0.82)	(0.05)
Treat × Mother’s education level (1 = junior high school and above)	0.21	0.13 **	−0.93	−0.02	1.14	0.07
	(1.03)	(0.06)	(1.10)	(0.07)	(1.08)	(0.06)
Adj. *R*^2^	0.08	0.05	0.10	0.07	0.08	0.05
*n*	801	801	802	802	802	802
Controls	Yes	Yes	Yes	Yes	Yes	Yes
Towns’ Fixed Effects	Yes	Yes	Yes	Yes	Yes	Yes

Data source: Authors’ survey. Note: The controls included the child’s age, gender, premature birth, number of siblings, breastfeeding, age of parents, parent’s education level, whether the mother was the primary caregiver, the ratio of the number of months the mother was at home full time to the child’s age, whether the daughter-in-law was not from the town, whether the parents were away from home, the grandmother’s rearing ability, caregivers’ ethnicity, and the family asset index. We also controlled separately for the level of mental health of the caregivers at baseline and the towns’ fixed effects. All standard errors account for clustering at the village level. ** *p* < 0.05.

**Table 7:** Program’s effect on the mental health of the daughters-in-law.

	Depression Scores (0–42)	Depressive Symptoms (1 = Yes)	Anxiety Scores (0–42)	Anxiety Symptoms (1 = Yes)	Stress Scores (0–42)	Stress Symptoms (1 = Yes)
	(1)	(2)	(3)	(4)	(5)	(6)
Panel A: Total sample of daughters-in-law						
Treatment (1 = yes)	−0.59	−0.07	−0.45	−0.06	−0.10	−0.06
	(0.46)	(0.04)	(0.50)	(0.04)	(0.55)	(0.04)
Adj. *R*^2^	0.12	0.06	0.10	0.07	0.08	0.04
*n*	562	562	562	562	562	562
Panel B: Daughters-in-law not from the town						
Treatment (1 = yes)	0.02	−0.02	−1.17	−0.15 **	−0.68	−0.11
	(0.74)	(0.06)	(0.89)	(0.07)	(1.13)	(0.07)
Adj. *R*^2^	0.16	0.06	0.14	0.10	0.09	0.05
*n*	197	197	197	197	197	197
Panel C: Daughters-in-law from the town						
Treatment (1 = yes)	−0.82	−0.09	−0.07	−0.02	0.16	−0.02
	(0.62)	(0.05)	(0.66)	(0.05)	(0.77)	(0.05)
Adj. *R*^2^	0.10	0.04	0.08	0.06	0.05	0.03
*n*	365	365	365	365	365	365
Controls	Yes	Yes	Yes	Yes	Yes	Yes
Towns’ Fixed Effects	Yes	Yes	Yes	Yes	Yes	Yes

Data source: Authors’ survey. Note: The controls included the child’s age, gender, premature birth, number of siblings, breastfeeding, age of parents, parent’s education level, the ratio of the number of months the mother was at home full time to the child’s age, whether the daughter-in-law was not from the town, whether the parents were away from home, the grandmother’s rearing ability, caregiver’s ethnicity, and the family asset index. We also controlled separately for the level of mental health of the caregivers at baseline and the towns’ fixed effects. All standard errors account for clustering at the village level. ** *p* < 0.05.

**Table 8:** Program effect on the social interactions of the daughters-in-law.

	Number of Friends Seen Regularly		Number of Times You Took Your Child to See Friends in the Past Month		Number of Families Communicated in the Past Month		Number of Days Communicated with Other Families Outside of Group Activities in the Past Month	
	DNT	DT	DNT	DT	DNT	DT	DNT	DT
	(1)	(2)	(3)	(4)	(5)	(60)	(7)	(8)
Treatment (1 = yes)	0.43	0.42	0.05	0.07	−0.18	0.13	12.78 **	18.30 **
	(0.43)	(0.31)	(0.15)	(0.11)	(0.36)	(0.34)	(2.37)	(2.20)
Adj. *R*^2^	0.19	0.09	0.04	0.08	0.13	0.21	0.22	0.27
*n*	198	365	197	364	180	332	180	332
Controls	Yes	Yes	Yes	Yes	Yes	Yes	Yes	Yes
Towns’ Fixed Effects	Yes	Yes	Yes	Yes	Yes	Yes	Yes	Yes

Data source: Authors’ survey. Note: The controls included the child’s age, gender, premature birth, number of siblings, breastfeeding, age of parents, parent’s education level, the ratio of the number of months the mother was at home full time to the child’s age, whether the daughter-in-law was not from the town, whether the parents were away from home, the grandmother’s rearing ability, caregivers’ ethnicity, and the family asset index. We also controlled the number of friends seen regularly and the number of times the caregivers took their child to see friends in the past month at baseline. Additionally, we controlled for the towns’ fixed effects. All standard errors account for clustering at the village level. ** *p* < 0.05. DT, daughter-in-law from the town; DNT, daughter-in-law not from the town.

**Table 9:** Mechanisms of the impact of the intervention on the mental health of female caregivers.

	Family Support		Parenting Behavior							
	Family Members Always Support Each Other (1 = yes)	Family Members Always Get Along with Each Other (1 = yes)	Read Books with Baby for the Past Three Days (1 = yes)	Told Stories to the Baby for the Past Three Days (1 = yes)	Sung Songs to the Baby for the Past Three Days (1 = yes)	Played Games with the Baby for the Past Three Days (1 = yes)	Named Things, Counted or Drew to Baby for the Past Three Days (1 = yes)	Number of Times That Yelled at the Baby Yesterday	Number of Times That Hit the Baby Yesterday	Explain Why His Behaviour Is Inappropriate When Disciplining the Baby (1 = yes)
Treatment (1 = yes)	0.06 **	0.02	0.16 **	0.09 **	0.13 **	0.17 **	0.12 **	−0.22	−0.13 **	−0.06 **
	−0.02	−0.02	−0.04	−0.03	−0.04	−0.04	−0.04	−0.15	−0.06	−0.02
Adj. *R^2^*	0.04	0.06	0.13	0.09	0.11	0.11	0.06	0.04	0.02	0.09
*n*	803	803	802	802	802	802	802	802	802	801
Controls	Yes	Yes	Yes	Yes	Yes	Yes	Yes	Yes	Yes	Yes
Tested Fixed Effects	Yes	Yes	Yes	Yes	Yes	Yes	Yes	Yes	Yes	Yes
	**Parenting Burden**				**Social Interaction (in the Past Month)**					
	**Baby Has Had a Fever for the Past Two Weeks (1 = yes)**	**Baby Coughing in the Past Two Weeks (1 = yes)**	**Number of Times the Baby Has Been Sick in the Past Two Weeks**		**Number of Friends Seen Regularly**	**Number of Times You Took Your Child to See Friends**		**Number of Families Communicated**	**Number of Days Communicated with Other Families Outside of Group Activities**	
Treatment (1 = yes)	0.01	−0.02	0.02		0.23	0.1		0.05	15.50 **	
	−0.03	−0.02	−0.04		−0.23	−0.07		−0.28	−1.49	
Adj. *R^2^*	0.01	0	0.01		0.08	0.05		0.17	0.24	
*n*	803	803	803		802	801		734	734	
Controls	Yes	Yes	Yes		Yes	Yes		Yes	Yes	
Tested Fixed Effects	Yes	Yes	Yes		Yes	Yes		Yes	Yes	

Note: The data source is the author’s survey. Controls include the child’s age, gender, premature birth, number of siblings, whether breastfeeding, age of parents, parent’s education level, whether the mother was the primary caregiver, the ratio of the number of months the mother was at home full time to the child’s age, whether daughter-in-law not from the town, whether parents were away from home, grandmother’s rearing ability, caregivers’ ethnicity, and family asset index. We also controlled separately for family support, parenting behavior, parenting burden, and social interaction at baseline and towns’ fixed effects. All standard errors account for clustering at the village level. ** *p* < 0.05. When regressions were performed on subsamples, whether the mother was the primary caregiver was omitted. The intervention significantly increased the grandmothers’ symptoms of depression, and we offer three possible explanations for this. First, older caregivers such as grandmothers suffer from depression more often and more severely than younger mothers [31,49]. In addition, this project increased the social interaction of the caregivers through group activities, and the depressed grandmother caregivers may have negatively influenced the non-depressed grandmother caregivers when communicating with them. Moreover, the intervention further reduced the grandmothers’ leisure breaks on top of the leisure time taken up by raising grandchildren [78], which may have increased their depressive symptoms. The lack of scientific parenting knowledge and feeding behaviors among the primary caregivers of children in poor rural areas of China [79] is also a possible explanation. Although the early childhood development intervention provided caregivers with scientific knowledge of feeding and parenting, the grandmothers may have further developed depressive psychological states in the face of the new knowledge they needed to learn and after learning about previous unscientific feeding and parenting practices.

## Data Availability

The data that support the findings of this study are available from the first author (Y.B.), upon reasonable request.

## Appendix A

**Table A1:** Balance test of the remaining sample after both attrition and exclusion.

	Full Sample	Treatment Group	Control Group	Difference: (2)−(3)
	Mean	Mean	Mean	*p*-Value
	(SD)	(SD)	(SD)	
	(1)	(2)	(3)	(4)
Child Characteristics				
Age (in months) (*n* = 821)	15.13	15.29	14.97	0.42
	(5.60)	(5.78)	(5.43)	
Male (1 = yes) (*n* = 821)	0.51	0.53	0.48	0.12
	(0.50)	(0.50)	(0.50)	
Premature (1 = yes) (*n* = 821)	0.05	0.04	0.05	0.32
	(0.21)	(0.19)	(0.23)	
Number of siblings (*n* = 821)	1.06	1.04	1.09	0.54
	(0.98)	(0.94)	(1.00)	
Breastfed (1 = yes) (*n* = 821)	0.96	0.96	0.97	0.47
	(0.19)	(0.21)	(0.18)	
Household Characteristics				
Mother’s age (*n* = 821)	26.65	26.88	26.41	0.24
	(5.74)	(5.84)	(5.64)	
Father’s age (*n* = 814)	29.73	29.67	29.78	0.78
	(5.48)	(5.41)	(5.56)	
Mother’s education level (1= junior high school and above) (*n* = 817)	0.49	0.49	0.50	0.86
	(0.50)	(0.50)	(0.50)	
Father’s education level (1= junior high school and above) (*n* = 813)	0.45	0.48	0.42	0.10
	(0.50)	(0.50)	(0.49)	
Primary caregiver (1 = mother) (*n* = 821)	0.82	0.82	0.83	0.70
	(0.38)	(0.39)	(0.38)	
The ratio of the number of months the mother was at home full time to the child’s age (*n* = 821)	0.92	0.93	0.92	0.60
	(0.20)	(0.19)	(0.20)	
Daughter-in-law not from the town (1 = yes) (*n* = 821)	0.36	0.35	0.37	0.53
	(0.48)	(0.48)	(0.48)	
Parents away from home (1 = yes) (*n* = 811)	0.56	0.55	0.56	0.72
	(0.50)	(0.50)	(0.50)	
Grandmother’s rearing ability (1 = yes) (*n* = 821)	0.65	0.64	0.66	0.64
	(0.48)	(0.48)	(0.48)	
Ethnicity (1 = Han) (*n* = 821)	0.92	0.94	0.90	0.04
	(0.28)	(0.24)	(0.30)	
Family asset index (*n* = 821)	0.00	0.02	–0.02	0.55
	(1.11)	(1.14)	(1.09)	
Caregiver’s Mental Health				
Depression symptoms (1 = yes) (*n* = 988)	0.32	0.33	0.31	0.71
	(0.47)	(0.47)	(0.46)	
Anxiety symptoms (1 = yes) (*n* = 989)	0.42	0.46	0.39	0.04
	(0.49)	(0.50)	(0.49)	
Stress symptoms (1 = yes) (*n* = 989)	0.29	0.28	0.31	0.29
	(0.46)	(0.45)	(0.46)	

Data source: Authors’ survey. Note: The family asset index was generated by the internationally used principal component analysis method based on the following six questions: Do you have a flush toilet in your home? Do you have a water heater at home? Do you have a computer at home? Do you have Internet access at home? Do you have air conditioning at home? Do you have a car/van at home? (The answer options for all questions are 1 = yes, 0 = no).

**Table A2:** Local average treatment effect on the mental health of the caregivers who participated in group activities nine times or more.

	Depression Scores (0–42)	Depressive Symptoms (1 = Yes)	Anxiety Scores (0–42)	Anxiety Symptoms (1 = Yes)	Stress Scores (0–42)	Stress Symptoms (1 = Yes)
	(1)	(2)	(3)	(4)	(5)	(6)
	Panel A: All samples					
Treatment (1 = yes)	0.01	–0.04	–0.01	–0.07	–0.16	–0.08
	(0.68)	(0.05)	(0.85)	(0.06)	(0.84)	(0.05)
Adj. *R*^2^	0.09	0.04	0.10	0.07	0.08	0.05
*n*	801	801	802	802	802	802
	Panel B: Mother subsamples					
Treatment (1 = yes)	–0.89	–0.10	–0.68	–0.08	–0.14	–0.08
	(0.70)	(0.06)	(0.75)	(0.06)	(0.80)	(0.06)
Adj. *R*^2^	0.11	0.05	0.10	0.07	0.08	0.04
*n*	562	562	562	562	562	562
	Panel C: Grandmother subsamples					
Treatment (1 = yes)	4.02	0.38 **	5.17	0.13	4.69	0.12
	(2.29)	(0.13)	(3.26)	(0.15)	(2.44)	(0.13)
Adj. *R*^2^	–0.00	0.07	0.07	0.11	0.21	0.11
*n*	88	88	89	89	89	89
Controls	Yes	Yes	Yes	Yes	Yes	Yes
Towns’ Fixed Effects	Yes	Yes	Yes	Yes	Yes	Yes

Data source: Authors’ survey. Note: The controls included the child’s age, gender, premature birth, number of siblings, breastfeeding, age of parents, parent’s education level, whether the mother was the primary caregiver, the ratio of the number of months the mother was at home full time to the child’s age, whether the daughter-in-law was not from the town, whether the parents were away from home, the grandmother’s rearing ability, caregivers’ ethnicity, and the family asset index. We also controlled separately for the level of mental health of caregivers at baseline and the towns’ fixed effects. All standard errors account for clustering at the village level. ** *p* < 0.05. When regressions were performed on the subsamples, whether the mother was the primary caregiver was omitted.

**Table A3:** Local average treatment effect on the mental health of the caregivers who participated in home visiting activities 22 times or more.

	Depression Scores (0–42)	Depressive Symptoms (1 = Yes)	Anxiety Scores (0–42)	Anxiety Symptoms (1 = Yes)	Stress Scores (0–42)	Stress Symptoms (1 = Yes)
	(1)	(2)	(3)	(4)	(5)	(6)
	Panel A: All samples					
Treatment (1 = yes)	0.01	–0.05	–0.01	–0.08	–0.18	–0.09
	(0.79)	(0.06)	(0.98)	(0.07)	(0.97)	(0.06)
Adj. *R*^2^	0.09	0.04	0.10	0.07	0.08	0.06
*n*	801	801	802	802	802	802
	Panel B: Mother subsamples					
Treatment (1 = yes)	–1.07	–0.12	–0.81	–0.10	–0.17	–0.10
	(0.83)	(0.07)	(0.88)	(0.07)	(0.97)	(0.07)
Adj. *R*^2^	0.12	0.06	0.10	0.08	0.08	0.05
*n*	562	562	562	562	562	562
	Panel C: Grandmother subsamples					
Treatment (1 = yes)	4.17	0.39 **	5.33	0.13	4.82	0.13
	(2.51)	(0.14)	(3.58)	(0.15)	(2.65)	(0.14)
Adj. *R*^2^	–0.05	0.01	0.02	0.09	0.15	0.09
*n*	88	88	89	89	89	89
Controls	Yes	Yes	Yes	Yes	Yes	Yes
Towns’ Fixed Effects	Yes	Yes	Yes	Yes	Yes	Yes

Data source: Authors’ survey. Note: The controls included the child’s age, gender, premature birth, number of siblings, breastfeeding, age of parents, parent’s education level, whether the mother was the primary caregiver, the ratio of the number of months the mother was at home full time to the child’s age, whether the daughter-in-law was not from the town, whether the parents were away from home, the grandmother’s rearing ability, caregivers’ ethnicity, and the family asset index. We also controlled separately for the level of mental health of the caregivers at baseline and the towns’ fixed effects. All standard errors account for clustering at the village level. ** *p* < 0.05. When regressions were performed on the subsamples, whether the mother was the primary caregiver was omitted.
